# Important contributors to foot pain at work and subsequent footwear considerations

**DOI:** 10.1080/19424280.2025.2493293

**Published:** 2025-06-20

**Authors:** Max Lewin, Roozbeh Naemi, Carina Price

**Affiliations:** School of Health Sciences, The University of Salford, Manchester, UK

**Keywords:** Foot pain, foot biomechanics, neural network, footwear, occupational standing

## Introduction

Occupational standing imposes substantial demands on the lower limbs of individuals at work. Clear links exist between prolonged standing at work and poor musculoskeletal (MSK) health including symptoms in the lower back, and lower extremity, alongside subjective discomfort and cardiovascular complications. Foot pain is also commonplace in workers with a high standing demand such as nurses and catering staff. Pain is limiting and contributes to presenteeism and absenteeism.

Footwear can modify forces between the floor and foot, attempting to manage loads during prolonged standing (Lewin & Price, 2024). Despite the role that footwear can play in standing roles, footwear is frequently deemed not fit for purpose by workers in occupations with specific requirements (Anderson et al., 2017). Understanding the role footwear in reducing pain or discomfort in standing workers is key to offer interventions to support these occupations.

## Purpose of the study

This research aimed to investigate the association between self-reported foot pain and footwear comfort, physical measures associated with foot characteristics and plantar pressure, and pain thresholds.

## Methods

Favourable opinion from the University ethics committee was gained (School of Health and Society HSR1819-055), employers provided access to their staff and workplace and chefs provided written informed consent.

Forty-Nine chefs (7 females, 42 males) (Height = 1.75 m ± 0.08, Mass = 82.6 kg ± 23.6, Age = 29 years ± 9.3) (Mean ± SD), participated in the research study. Participants engaged in an hour-long data collection at their workplaces.

The foot health status questionnaire (FHSQ) was used to quantify foot pain over the previous two weeks. Foot posture index (FPI) was measured by an experienced rater.

In-shoe plantar pressure measurement was completed using the Pedar-X system (Novel Gmbh, Munich, Germany), during a sixty second dynamic standing protocol. Plantar pressure was exported with seven regions of interest (ROI) defined in previous research using Matlab (Natick, Massachusetts, USA) (Lewin & Price, 2024). Peak pressure and contact area were exported and averaged for each ROI from 10 second windows of the 60 second trial.

Participants wore their own work footwear and rated its comfort using a 100 mm visual analogue scale (VAS).

Pain-pressure threshold was measured at 6 regions (Heel, lateral midfoot, medial midfoot, MH1, MH3, MH5) using the ForceTen algometer (Wagner instruments, Connecticut, USA), and subsequently averaged across the locations.

Workplace standing time was measured using Activpal accelerometers (pal technologies, Glasgow, Scotland).

A Multilayer Perception Neural Network model with a single hidden layer was utilised to describe FHSQ foot pain as the dependent variable. In this model FPI was used as a factor, and MH1 PP, Medial midfoot contact area, shoe comfort, Body Mass Index (BMI), standing time, and pain-pressure threshold were covariates. Variables were included in the neural network model based on an unpublished systematic review.

## Results

Average data and importance values are presented in Table 1.

**Table 1.** Variable measures and importance values from the Neural Network model.

	Mean ± SD	Importance
FHSQ foot pain	71.1 ± 22.2	
MH1 PP (kPa)	62.4 ± 27.6	0.309
FPI	2.96 ± 1.72	0.144
Pain-Pressure Threshold (kPa)	357 ± 165	0.133
BMI (kg/m^2^)	27.01 ± 6.97	0.126
Shoe Comfort	61.2 ± 21.7	0.113
MM CA (cm^2^)	11.5 ± 6.65	0.090
Standing time (minutes)	438 ± 112	0.087

All participants were entered into the Neural Network model with 37 used to train and 12 to test the model. The sum of squares error was 3.767.

## Discussion and conclusion

The ANN identified MH1 peak pressure to be the most important factor to foot pain, with time spent standing at work the least important.

The results may indicate that footwear-based interventions for reducing MH1 peak pressure could reduce reported foot pain at work. Further important considerations include foot posture, and pain-pressure threshold. Other factors such as BMI should be considered by the individual.

Footwear considerations can be made to address differences in foot posture, sensitivity, and changes associated with higher BMI such as greater load and foot shape.
